# Practice patterns in the diagnosis and management of chemotherapy-induced peripheral neuropathy in adolescents and young adults with cancer: a survey of oncologists

**DOI:** 10.1007/s00520-025-09387-9

**Published:** 2025-04-04

**Authors:** Jennifer A. Belsky, L. Lee Dupuis, Lillian Sung, Allie Carter, Audrey Leisinger, Etan Orgel, Susan K. Parsons, Michael Roth

**Affiliations:** 1 Department of Pediatrics, Indiana University School of Medicine, Indianapolis, USA; 2 Department of Pharmacy, Leslie Dan Faculty of Pharmacy, Research Institute, the Hospital for Sick Children, University of Toronto, Toronto, Canada; 3 Division of Haematology/Oncology, The Hospital for Sick Children, Toronto, Canada; 4 Department of Biostatistics and Health Data Science, Indiana University School of Medicine, Indianapolis, USA; 5 Cancer and Blood Disease Institute, Children’s Hospital los Angeles, Los Angeles, USA; 6 Institute for Clinical Research and Health Policy Studies, Tufts Medical Center, Reid R. Sacco AYA Cancer Program, Boston, USA; 7 MD Anderson Cancer Center, University of Texas, Austin, USA

**Keywords:** Peripheral neuropathy, Adolescent young adult, Supportive care, Pediatric oncology

## Abstract

**Purpose:**

Chemotherapy-induced peripheral neuropathy (CIPN) affects > 78% of oncology patients and causes detrimental side effects. There may be practice heterogenicity in CIPN management amongst oncologists treating pediatric, adolescent young adult (AYA), and adult patients with cancer. We sought to evaluate the practice patterns of oncologists regarding their management of CIPN in AYAs with cancer.

**Methods:**

A survey was developed and sent to pediatric and medical oncologists from across the United States. Scenarios included an 18-year-old receiving vincristine (VCR) with mild neuropathy (Scenario 1) and moderate/severe neuropathy (Scenario 2). Respondents were asked how they would manage each patient. Differences between pediatric and medical oncologists’ management were assessed.

**Results:**

A total of 179 responses were submitted by 132 (73.7%) pediatric, 44 (24.6%) medical oncologists, and 3 (1.6%) oncologists who care for both pediatric and adult patients. Over half of respondents for Scenario 1 would refer the patient to physical therapy (PT) (56.8%), 38.1% would prescribe a pharmacologic agent, and 27.8% would dose reduce/omit vincristine. For Scenario 2, most (81.8%) would dose reduce/omit vincristine, 69.3% would refer for PT, and 44.9% would start a pharmacologic agent. On multivariable analyses, medical oncologists were more likely to dose reduce/omit vincristine for Scenario 1 (OR, 7.68; 95% CI, 3.24–18.22) and Scenario 2 (OR, 5.52; 95% CI, 1.37–22.18, and less likely to refer to PT for Scenario 1 (OR, 0.12; 95% CI, 0.05–0.31) and Scenario 2 (OR, 0.18; 95% CI, 0.08–0.41).

**Conclusion:**

Our survey suggests a broad spectrum of CIPN management in AYAs with cancer. The heterogenicity in practices and significant differences between pediatric and medical oncologists underscores an urgent need to better understand the source of heterogeneity in CIPN management practices and barriers to evidence-based care delivery.

**Supplementary Information:**

The online version contains supplementary material available at 10.1007/s00520-025-09387-9.

## Background

Survival for children, adolescents, and young adults (AYAs, age 15–39 years at diagnosis) and older adults with cancer has improved meaningfully over time [1]. Although many patients are cured of their disease, most suffer from chemotherapy-induced side effects during therapy and into survivorship [2]. Chemotherapy-induced peripheral neuropathy (CIPN) is a common chemotherapy treatment side effect, affecting up to 78% of patients receiving neurotoxic chemotherapy [3, 4]. It is characterized by symptoms including sensory, motor, and/or autonomic dysfunction, most notably leading to extremity pain and weakness, impaired tendon reflexes, balance and vibration senses, gait disturbances, and constipation [4]. These toxicities can be both acute and chronic, the latter resulting in long-lasting effects on physical function, school and work performance, and health-related quality of life [5]. Recent studies have reported that 82% of children and AYAs had newly identified CIPN at the end of their routine treatment plan, suggesting that their symptoms may not have been appropriately diagnosed or managed while receiving chemotherapy [6].

Management of CIPN has proven to be challenging across all patient populations. Existing clinical practice guidelines for neuropathy all apply to adult cancer survivors with CIPN [7] or adults with generalized chronic neuropathic conditions not caused by chemotherapy and with other comorbid conditions [7] [8]. No guidance regarding CIPN prevention is available. For CIPN treatment, ASCO recommends duloxetine for adult cancer survivors with painful CIPN (Recommendation strength: moderate; Evidence quality: intermediate) [7]. The ESMO guidance document also recommends duloxetine for adults with neuropathic pain (Grade of recommendation: Moderate to strong; Level of evidence: at least 1 large randomized controlled trial or meta-analysis available) [8]. NICE recommends amitriptyline, duloxetine, gabapentin, or pregabalin for the initial treatment of neuropathic pain other than trigeminal neuralgia in adults or capsaicin cream for those with localized neuropathic pain [9]. The NCCN’s guidance for adult cancer pain includes a brief list of possible treatment options for neuropathic pain including antidepressants, anticonvulsants, topical agents, and Interventional Strategies [10]. There is no guidance regarding the management of CIPN that is specific to the pediatric or AYA settings.

AYAs are a unique group of patients who are cared for by both pediatric and medical oncologists across a variety of clinical settings [11]. The availability of limited guidance for managing both acute and long-term CIPN in this challenging patient population suggests that the care of these patients may be variable between institutions and physicians. No studies have assessed the practice patterns of either pediatric or medical oncologists for CIPN management of AYAs. Since investigating the possible heterogeneity in practice patterns of both pediatric and medical oncologists is imperative to inform future trials to optimize CIPN treatment in these patients, we undertook a national, scenario-based survey of medical and pediatric oncologists to describe their approaches to the management of CIPN and to compare approaches taken by medical and pediatric oncologists.

## Methods

We conducted a national scenario-based survey of medical and pediatric oncologists to describe CIPN management practices. This survey was approved by the institutional review board at Indiana University, and the requirement for consent was waived.

### Survey development

Survey questions were developed by the investigators using a Research Electronic Data Capture™ (REDCap™) database, and refined iteratively [12]. The survey was piloted by the primary investigator at Indiana University School of Medicine with a panel of 10 pediatric and medical oncologists, and the survey was revised according to this feedback and finalized.

**Fig. 1:**
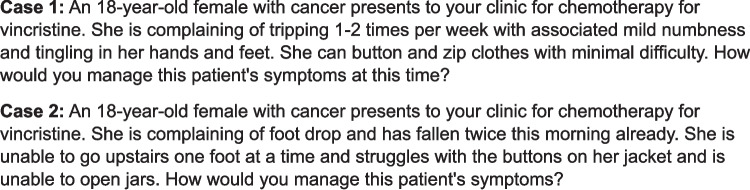
Scenario descriptions. Case 1: An 18-year-old female with cancer presents to your clinic for chemotherapy for vincristine. She is complaining of tripping 1–2 times per week with associated mild numbness and tingling in her hands and feet. She can button and zip clothes with minimal difficulty. How would you manage this patient’s symptoms at this time? Case 2: An 18-year-old female with cancer presents to your clinic for chemotherapy for vincristine. She is complaining of foot drop and has fallen twice this morning already. She is unable to go upstairs one foot at a time and struggles with the buttons on her jacket and is unable to open jars. How would you manage this patient’s symptoms?

Survey questions referred to two scenarios: an 18-year-old receiving vincristine (VCR) with mild neuropathy (Scenario 1) and with moderate/severe neuropathy (Scenario 2) (Fig. 1).

Oncologists could select more than one option for each response for each case-based scenario; however, only one response option was allowed for subsequent questions. Additional information related to respondent characteristics (pediatric or medical oncologist, sex, years of practice, patient population cared for, and practice setting) and general CIPN management (pharmacologic management, chemotherapy reduction, and physical therapy referrals) was collected. A full list of survey questions is available in the Supplemental Material.

### Survey distribution

Eligibility criteria included being a practicing pediatric or medical oncologist in the United States. Email addresses of oncologists were obtained from hospital websites and other publicly available online websites for physicians practicing in the United States at academic and community cancer centers. The survey link was distributed via email to both pediatric and medical oncologists within the United States at two separate time points, 8 weeks apart over the four-month study period. The survey was available from June 2023 to September 2023. Responses were anonymous and securely stored in a REDCap™ database through the Indiana University School of Medicine.

### Data analysis

Analyses of specific scenario responses were limited to respondents who identified as medical or pediatric oncologists and responses of individuals who self-identified as other provider types by survey questions were excluded. Survey responses were categorized by oncologist type (medical vs pediatric) and described as frequencies. Categorical responses and demographics were compared between oncologist types by the Chi-Square test or Fisher’s Exact test and ordinal responses and demographics were compared by the Mantel–Haenszel test. Multivariable models were performed for the effect of oncologist type on different outcomes including predictors for sex, years of practice, and practice setting (academic vs other) to identify possible confounding variables using fourth logistic regression. These variables were chosen post hoc as they were the respondent characteristics that were significantly associated with the oncologist type, while the practice setting was chosen over the other center characteristics due to the higher statistical significance. For each subject, responses to Scenario 1 were compared to responses to Scenario 2 with McNemar’s test. Data summaries were produced using R Statistical Software (version 4.1.0). All analyses were performed using SAS version 9.4 (SAS Institute, Inc., Cary, NC, USA).

## Results

The survey link was sent to 1351 oncologists; of which 246 were undeliverable. A total of 185 responses were received including six responses from non-oncologists who were omitted from the analysis. Thus, 179 responses were included in overall statistical analyses.

### Respondent demographics

Responses were submitted by 132 (73.7%) pediatric and 44 (24.6%) medical oncologists, with three (1.7%) caring for both patient populations. (Table 1). Participants had a wide range of years in practice (48.6% of respondents > 10 years) and the majority worked in an academic medical center (72.1%).

**Table 1:** Characteristics of # oncologist survey respondents

Entire cohort	
**Characteristic**	***N* (%)**
Oncologist type	
Pediatric oncologist	132 (73.7)
Medical oncologist	44 (24.6)
Both pediatric and medical oncologist	3 (1.68)
Sex, female	104 (58.1)
Years of practice after fellowship	
< 5 years	54 (30.2)
5–9 years	38 (21.2)
10–19 years	47 (26.3)
20–30 years	23 (12.9)
> 30 years	17 (9.5)
Patient cohort cared for	
Leukemia	105 (58.7)
Lymphoma	90 (50.3)
Solid tumor	91 (50.8)
Neuro-oncology	36 (20.1)

### Management of mild CIPN

**Fig. 2:**
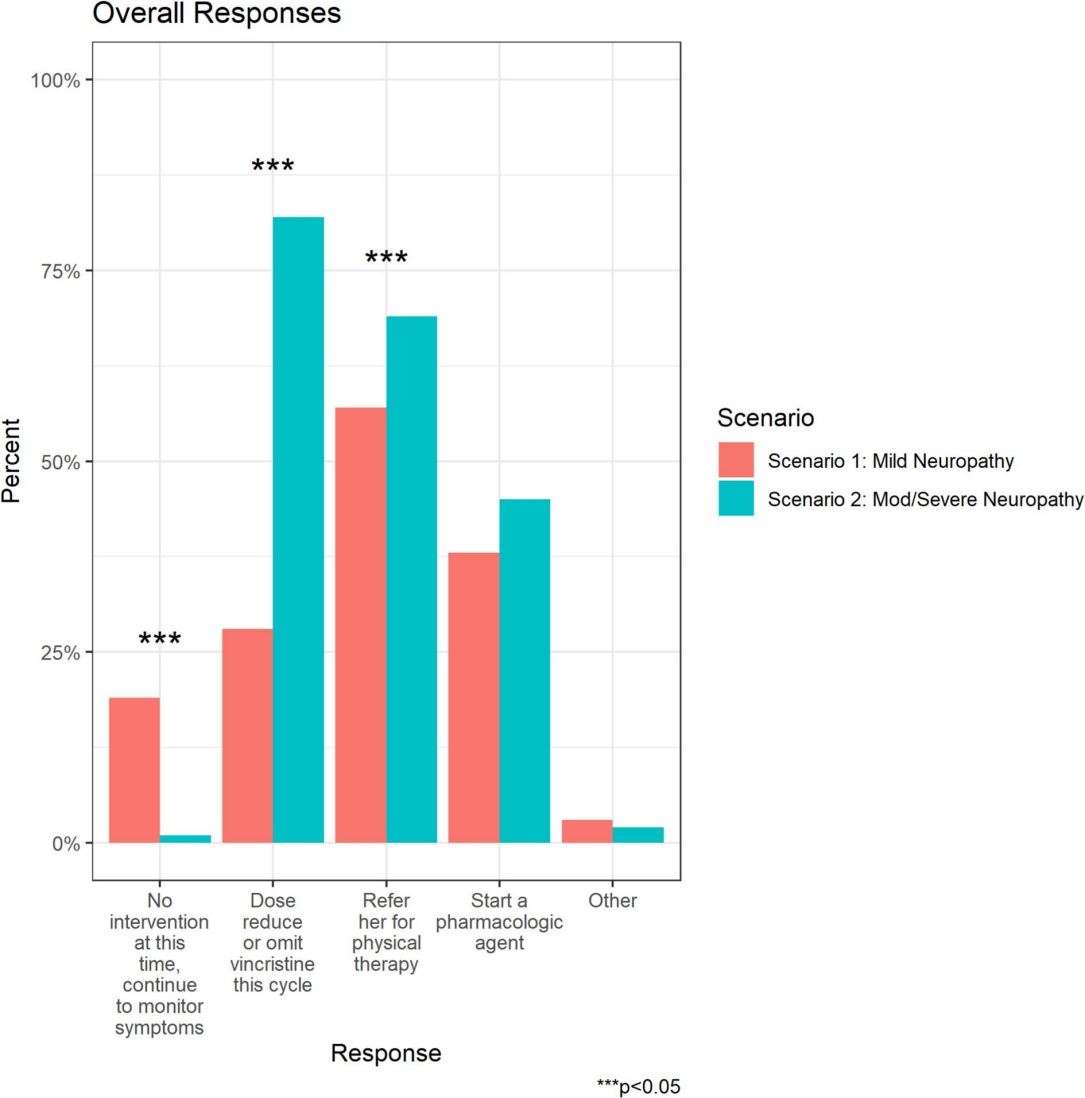

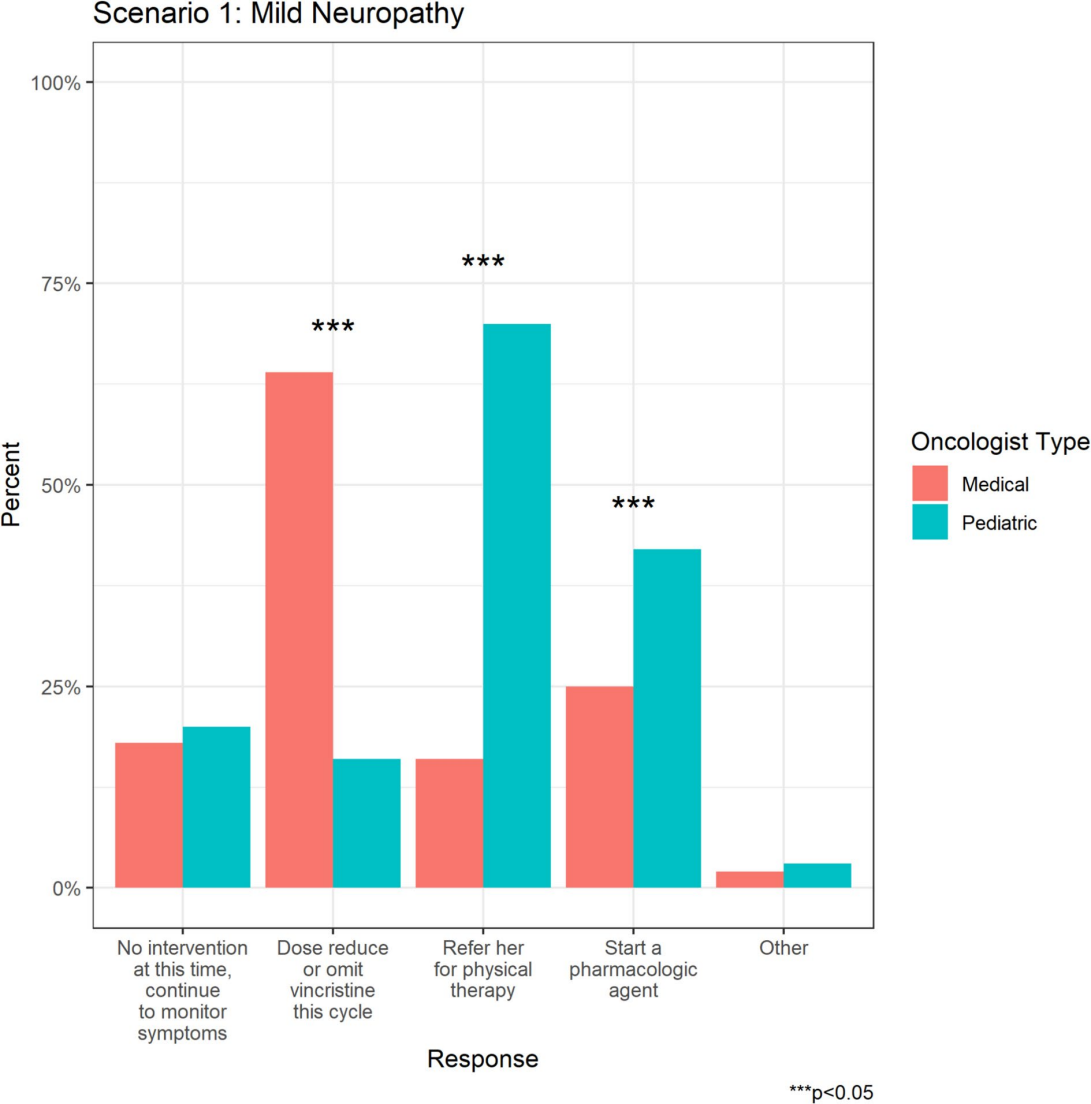

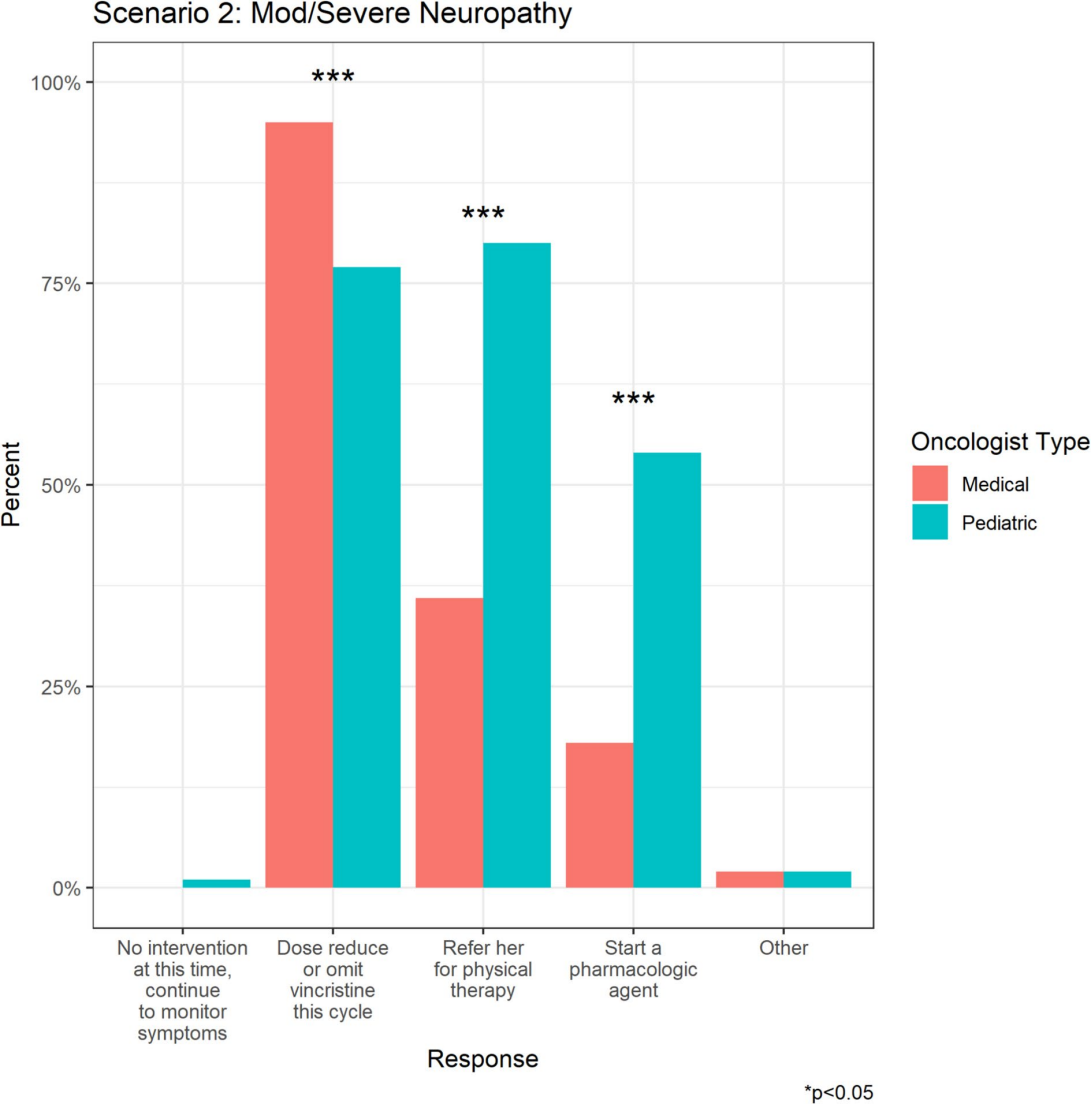
Nnnn **a** Overall oncologist management responses for CIPN in Scenarios 1 and 2. **b** Differences in pediatric vs medical oncologist responses CIPN Scenario 1. **c** Differences in pediatric vs medical oncologist responses CIPN Scenario 2

Greater than half of respondents would refer the patient with mild CIPN to physical therapy (PT) (*n* = 100, 56.8%), while 67 (38.1%) would prescribe a pharmacologic agent, and 49 (27.8%) would dose reduce or omit vincristine. A further 34 respondents (19.3%) would monitor symptoms without intervention (Fig. 2a).

Medical oncologists were significantly more likely to dose reduce vincristine compared to pediatric oncologists (63.3% vs 15.9%, p < 0.001) whereas pediatric oncologists were more likely to refer the patient to PT (70.5% vs 15.9%, *p* < 0.001), and/or prescribe a pharmacologic agent (42.4% vs 25.0%, *p* = 0.039) (Fig. 2b).

**Fig. 3:**
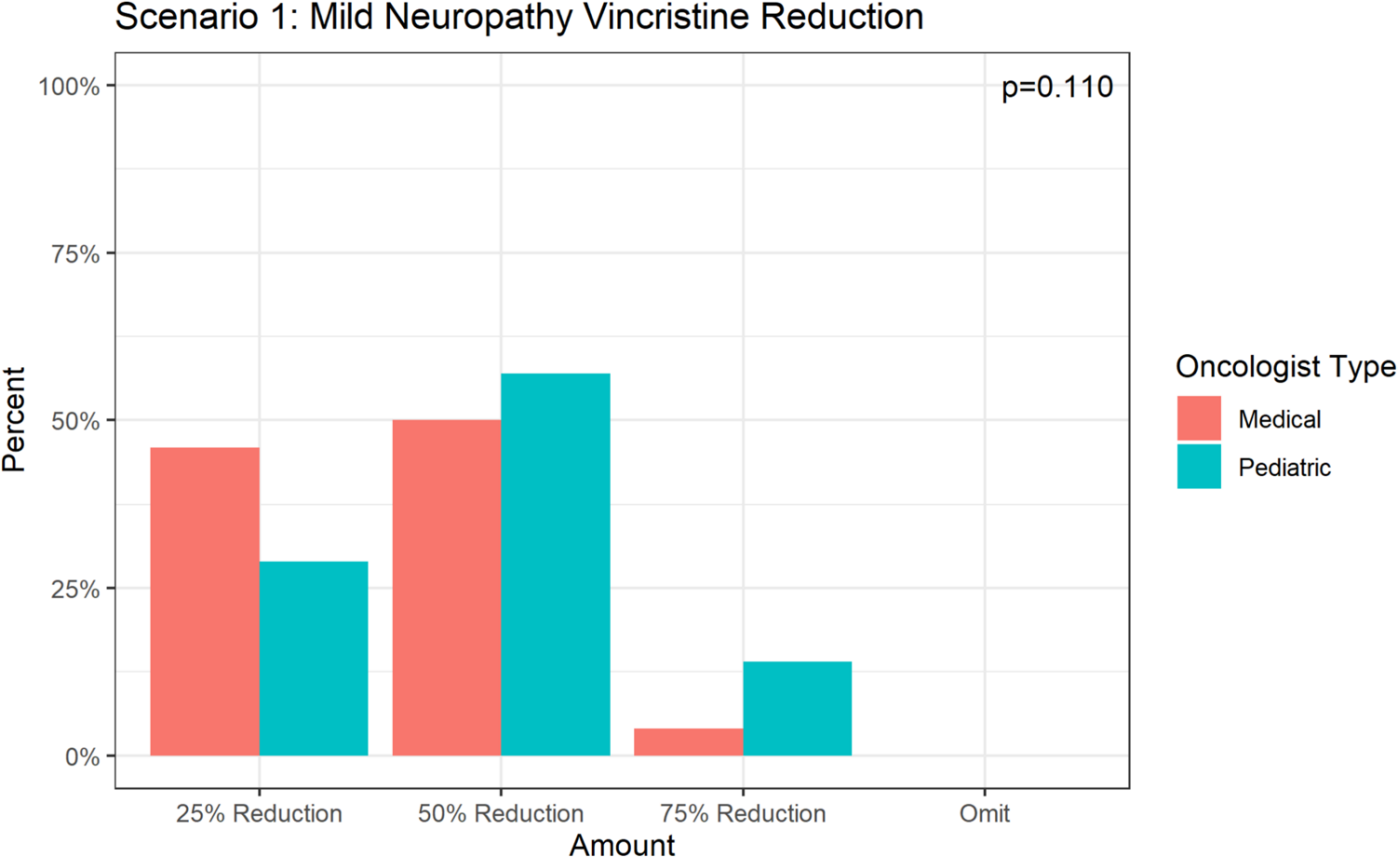

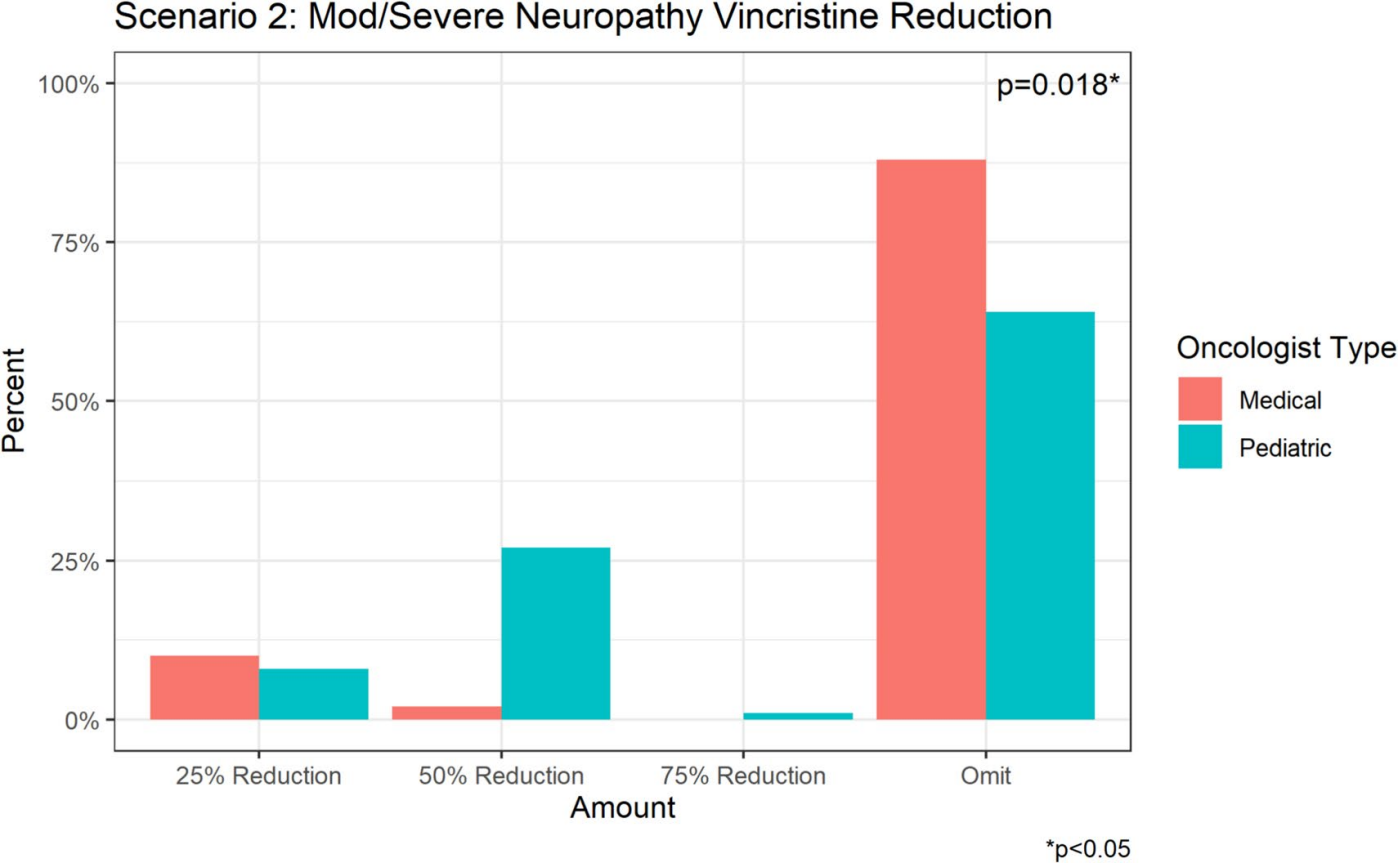
Variation in practice patterns regarding vincristine dose reduction in **a** Scenario 1 and **b** Scenario 2

Of the 50 respondents who chose vincristine dose reduction, approximately half (54.0%) selected a 50% dose reduction, 38.0% selected a 25% dose reduction, and 8.0% selected a 75% dose reduction (Fig. 3a). No respondents opted for dose omission (100% dose reduction).

### Management of moderate/severe CIPN

Most respondents (81.8%) would dose-reduce or omit vincristine, while 69.3% would refer to PT, and 44.9% would prescribe a pharmacologic agent (Fig. 2a). Medical oncologists were significantly more likely to dose-reduce vincristine compared to pediatric oncologists (95.5% vs 77.3%, *p* = 0.007). In contrast, pediatric oncologists were significantly more likely to refer the patient to PT (80.3% vs 36.4%, *p* < 0.001), and/or prescribe a pharmacologic agent (53.8% vs 18.2%, *p* < 0.001) (Fig. 2c).

Of the 147 (82.1%) respondents who chose vincristine dose reduction, most (70.8%) selected to omit the dose (100% dose reduction) while 20.4% selected a 50% dose reduction and 8.2% opted for a 25% dose reduction (Fig. 3b).

### Practice patterns regarding PT referral and initiation of pharmacotherapy

**Fig. 4:**
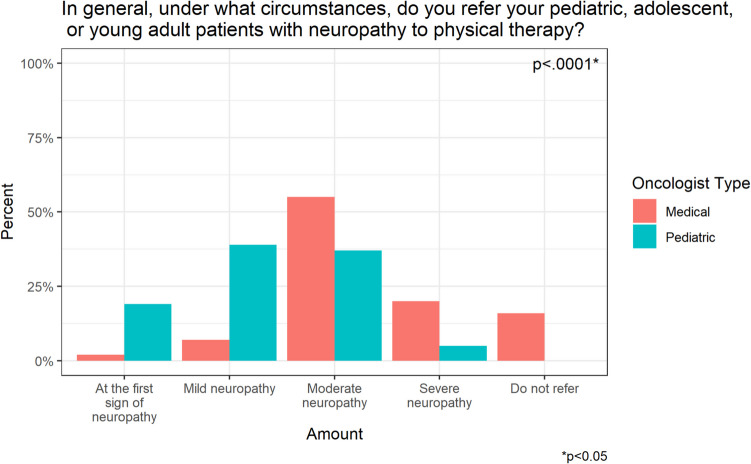

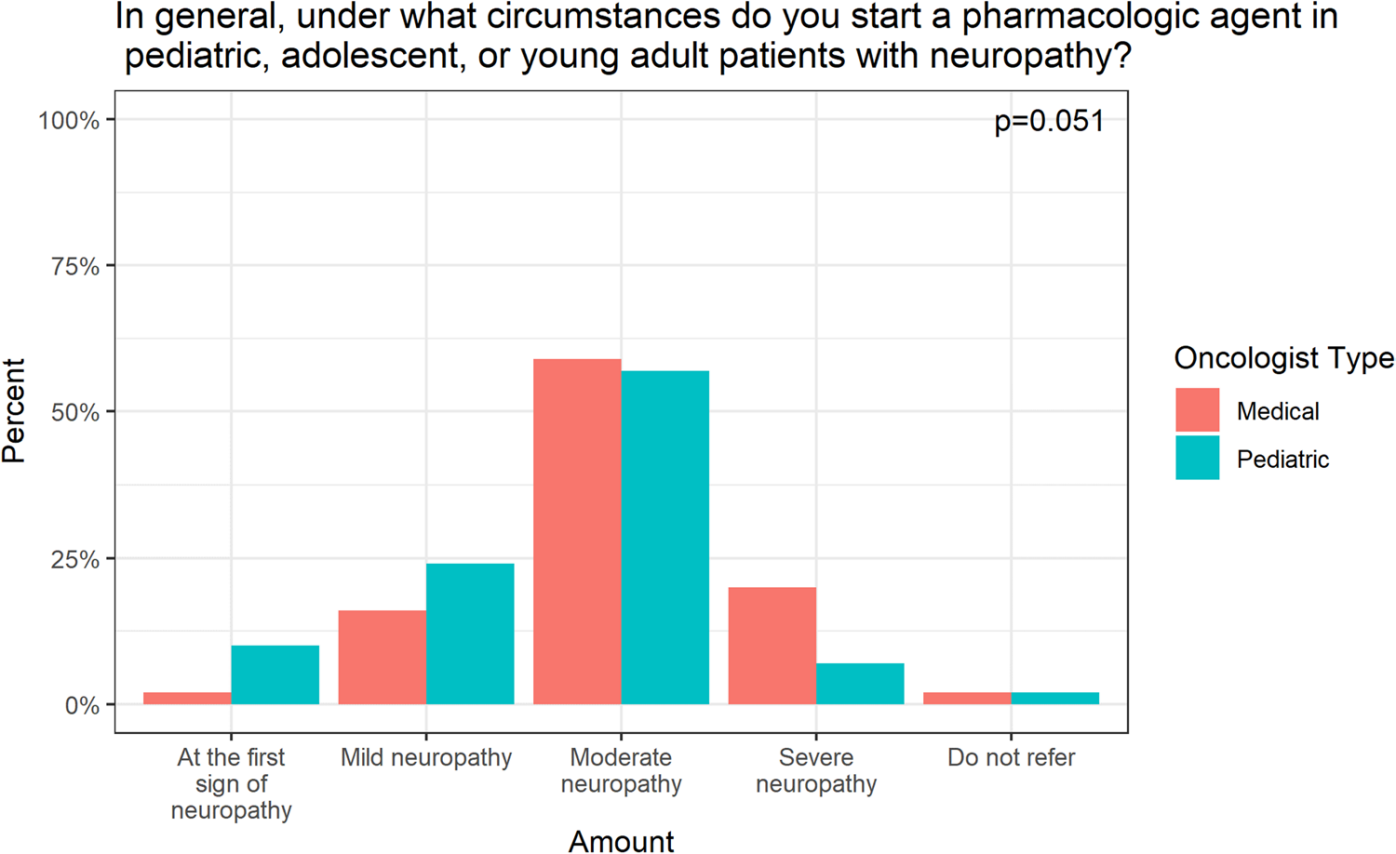
Respondents’ **a** physical therapy referral practice patterns and **b** respondents’ pharmacologic agent practice patterns

Oncologists would refer to PT at the first sign of neuropathy (*n* = 26, 14.5%), mild neuropathy (*n* = 55, 30.7%), moderate neuropathy (*n* = 76, 42.5%), and severe neuropathy (*n* = 15, 8.4%). Seven oncologists (3.9%) would never refer to PT (Fig. 4a).

Oncologists would initiate a pharmacologic agent at the first sign of neuropathy (*n* = 14, 7.8%), mild neuropathy (*n* = 39, 21.8%), moderate neuropathy (*n* = 103, 57.5%), and severe neuropathy (*n* = 19, 10.6%). Four respondents (2.2%) never initiated a pharmacologic agent (Fig. 4b). Among oncologists who would prescribe medications for CIPN, gabapentin was the most common (50.8%), followed by pregabalin (7.3%), topical treatments (4.47%) and amitriptyline (2.8%).

### Comparison of medical and pediatric oncologist approaches to CIPN management

On multivariable analyses (Table 2), adjusting for sex, years of practice, and practice setting, medical oncologists were more likely to dose reduce or omit vincristine for mild CIPN (OR, 7.68; 95% CI, 3.24–18.22) and moderate/severe CIPN (OR, 5.52; 95% CI, 1.37–22.18) and less likely to refer to PT for mild CIPN (OR, 0.12; 95% CI, 0.05–0.31) and moderate/severe CIPN (OR, 0.18; 95% CI, 0.08–0.41).

**Table 2:** Multivariable analyses response comparison: medical vs pediatric oncologists (*N* = 44 vs *N* = 132) adjusting for sex, years of practice, and practicing in an academic medical center (reference is a pediatric oncologist)

Outcome	Odds ratio estimate	95% confidence limits		Adjusted *p*-value
Mild CIPN (Scenario 1)				
Dose reduce or omit vincristine this cycle	7.68	3.24	18.22	< .0001
Refer to physical therapy	0.12	0.05	0.31	< .0001
Start a pharmacologic agent	0.50	0.21	1.17	0.109
Moderate/Severe CIPN (Scenario 2)				
Dose reduce or omit vincristine this cycle	5.52	1.37	22.18	0.016
Refer to physical therapy	0.18	0.08	0.41	< .0001
Start a pharmacologic agent	0.22	0.09	0.54	0.001

OR < 1 indicates a lower odds of the intervention among medical oncologists compared to pediatric oncologists

Among the respondents who chose to dose-reduce vincristine for a patient with mild CIPN, there was no statistically significant difference between the level of dose reduction (*p* = 0.110) between pediatric and medical oncologists. In the patient with moderate/severe CIPN, medical oncologists were significantly more likely to omit vincristine (88.1% vs 63.7%, *p* = 0.004), whereas pediatric oncologists were significantly more likely to dose reduce by 50% (27.5% vs 2.4%, *p* = 0.001). There was a significant difference in the circumstances under which medical vs pediatric oncologists would initiate a pharmacologic intervention (*p* = 0.01). Of the respondents, 24.2% of pediatric oncologists would initiate a pharmacologic intervention for a patient with mild neuropathy versus 15.9% of medical oncologists. Both pediatric (56.8%) and medical oncologists (59.1%) would initiate a pharmacologic intervention for a patient with moderate neuropathy. Lastly, 20.5% of medical oncologists reported that they would initiate a pharmacologic intervention for a patient with severe neuropathy, compared to 6.8% of pediatric oncologists.

## Discussion

We identified significant variability and heterogenicity in CIPN management among United States-based oncologists responding to a survey and differences in practice between pediatric and medical oncologists. Pediatric oncologists were more likely to refer AYAs with CIPN for PT and start a pharmacologic agent, whereas medical oncologists were more likely to dose reduce or omit vincristine. These differences in vincristine dose modification, medical management, and supportive care likely impact cancer-directed treatment and quality of life outcomes meaningfully.

Although CIPN symptoms may be partially reversible, neuropathic symptoms often persist throughout survivorship [13]. The impact of CIPN on patients’ ability to perform simple activities of daily living including typing, writing, walking stairs, buttoning clothes, and avoiding falls can result in decreased occupational and social role performance [14, 15]. Furthermore, many patients with severe CIPN report a sense of isolation, depression, and lost sense of purpose due to their symptoms [16, 17]. Evidence to support specific interventions to prevent and treat this important symptom in adult patients is limited and negligible in children. Further, guidance from ASCO, ESMO, and NCCN is not directly applicable to AYAs with CIPN. The highly variable management of CIPN observed in our survey may be due, in part, to the scarcity of direct evidence generally and the lack of guidance for AYAs actively receiving chemotherapy.

Our survey elicited significant and striking differences in responses regarding vincristine dose reduction strategies between pediatric and medical oncologists caring for patients with CIPN. Medical oncologists were significantly more likely to not only dose reduce vincristine in the setting of CIPN of any severity, but also more likely to omit the dose completely. The tolerance of symptoms and side effect management in medical compared to pediatric oncology practice may play a role in CIPN management. Prior studies have demonstrated that medical oncologists primarily recommend gabapentin for CIPN management despite adequate evidence, which is similar to our responses [18]. Furthermore, clinician perspective themes from prior work including utilizing non-recommended management strategies or lack of standardization for chemotherapy dose reductions are in keeping with our wide range of pharmacologic interventions and dose reduction strategies [19]. Medical oncologists likely care for an older patient population with a greater number of comorbid conditions of higher severity than pediatric oncologists. Thus, risks of morbidity and mortality with receipt of intensive chemotherapy may be higher in the patient cohort cared for by medical oncologists. This could contribute to our finding that medical oncologists were more likely to recommend earlier and more aggressive dose reductions.

Vinca alkaloids have long been a cornerstone of therapy in both the adult and pediatric patient populations to treat a majority of cancers. Large dose reductions or omissions in vinca alkaloid therapy have the potential to decrease overall survival in patients by reducing their overall chemotherapy exposure needed to cure their underlying cancer. In the pediatric and AYA setting, the Children’s Oncology Group phase 3 study AHOD1331 managed the emergence of CIPN following treatment with dual tubulin toxins (i.e., vincristine on day 1 and day 8 in the standard arm; brentuximab vedotin on day 1 and vincristine on day 8 in the experimental arm)[20]. Protocol-stipulated dose modification called for the reduction first of vincristine, and then, the experimental agent, Brentuximab vedotin, and found it did not impact progression-free survival[20]. No studies have investigated the impacts of sequential dose reductions or omissions of vinca alkaloids in adults with CIPN, particularly when more than one tubulin toxin is used. In addition, despite the limited evidence for the efficacy of pharmacotherapy in AYAs less than 18 years of age, pediatric oncologists were more likely to start a pharmacologic agent in either mild or moderate/severe scenarios as compared to their medical oncologist counterparts. Nevertheless, no respondent selected to initiate duloxetine, as recommended by ASCO and ESMO for adults with CIPN.

As with most surveys, using a broad sampling strategy, our results are limited by a likely overall low response rate with fewer medical oncologists responding compared with pediatric oncologists. Furthermore, only a single age was queried in our survey of an 18-year-old female. The AYA age range is broad, and with this heterogeneous population, it is unclear whether medical oncologists treat patients in their 30 s similarly to patients in their late teens. Of note, the survey only queried hypothetical action in response to emerging CIPN from a single agent (vincristine), which may not extend to management in the setting of dual tubular toxins, such as the aforementioned AHOD1331 study or the recently completed SWOG-led S1826 (NCT03907488) study (vinblastine and Brentuximab vedotin) [21]. Future studies should discern differences in CIPN management with regard to different neurotoxic agents. Due to survey confidentiality, while it is unlikely providers took the survey more than once, we cannot guarantee that there were no duplicate responses. The study asked respondents to share their recommended treatment approaches to two scenarios of AYAs with CIPN; however, it is not clear if oncologists’ real-world practices completely mirror their responses as a chart review or prospective study was not conducted. Despite these limitations, to our knowledge, this survey is the first to explore the real-life practice patterns of oncologists managing CIPN and the variability in practice between pediatric and medical oncologists.

CIPN is a frequent short- and long-term side effect of cancer treatment in AYAs. However, management of CIPN varies greatly overall and between medical and pediatric oncologists. This underscores an urgent need to better understand CIPN management practices and barriers to evidence-based care delivery. High-quality research is also needed to optimize CIPN management in patients with cancer.

## Supplementary Information

Below is the link to the electronic supplementary material.

Supplementary file1 (PDF 45 KB)

## Data Availability

No datasets were generated or analysed during the current study.
