# Supplementary material for: Practice patterns in the diagnosis and management of chemotherapy-induced peripheral neuropathy in adolescents and young adults with cancer: a survey of oncologists
Source: Support Care Cancer. 2025 Apr 4;33(4):350. doi: 10.1007/s00520-025-09387-9 (PMC11971165; doi:10.1007/s00520-025-09387-9)
Supplement: Supplementary file 1 — Supplementary file1 (PDF 45 KB) [file 520_2025_9387_MOESM1_ESM.pdf]

Neuropathy Practice Patterns of Pediatric and Medical Oncologists

Please complete the survey below.

Thank you!

An 18-year-old female with cancer presents to your clinic for chemotherapy for vincristine. She is complaining of tripping 1-2 times per week with associated mild numbness and tingling in her hands and feet. She can button and zip clothes with minimal difficulty.<br>How would you manage this patient's symptoms at this time? (select all that apply)	☐ No intervention at this time, continue to monitor symptoms<br>☐ Dose reduce or omit vincristine this cycle<br>☐ Refer her for physical therapy<br>☐ Start a pharmacologic agent<br>☐ Other
Please fill in other management option	__________________________________
If reduce or omit vincristine for the patient, what percentage would you reduce	◯ 25%<br>◯ 50%<br>◯ 75%
Please select the medications that you would prescribe	☐ Gabapentin (Neurotin)<br>☐ Pregabalin (Lyrica)<br>☐ Topical Treatment (capasaicin cream or lidocaine patches)<br>☐ Amitriptyline<br>☐ Other Pharmacologic agent
Please fill in other pharmacologic agent	__________________________________
An 18-year-old female with cancer presents to your clinic for chemotherapy for vincristine. She is complaining of foot drop and has fallen twice this morning already. She is unable to go upstairs one foot at a time and struggles with the buttons on her jacket and is unable to open jars.<br>How would you manage this patient's symptoms? (select all that apply)	☐ No intervention at this time, continue to monitor symptoms<br>☐ Dose reduce or omit vincristine this cycle<br>☐ Refer for physical therapy<br>☐ Start a pharmacologic agent (may select more than one)<br>☐ Other
Please fill in other management option	__________________________________
If you reduce or omit vincristine for the patient, what percentage would you reduce	◯ 25%<br>◯ 50%<br>◯ 75%<br>◯ Omit
Please select the medications that you would prescribe	☐ Gabapentin (Neurotin)<br>☐ Pregabalin (Lyrica)<br>☐ Topical Treatment (capasaicin cream or lidocaine patches)<br>☐ Amitriptyline<br>☐ Other Pharmacologic agent
Please fill in other pharmacologic agent	__________________________________

In general, for your patients receiving agents known to cause neuropathy, how often do you routinely ask your patients/caregivers about the following domains?

	Never	Rarely	Some Visits	Most Visits	Every Visit
balance	◯	◯	◯	◯	◯
tripping	◯	◯	◯	◯	◯
numbness	◯	◯	◯	◯	◯
tingling	◯	◯	◯	◯	◯
constipation	◯	◯	◯	◯	◯
grip strength	◯	◯	◯	◯	◯
hand cramping	◯	◯	◯	◯	◯
ability to manage buttons and/or zippers	◯	◯	◯	◯	◯

Are there other questions or information you ask your patients/caregivers about neuropathy?
____

Do you utilize any validated patient-reported outcome measures to assess peripheral neuropathy in your pediatric, adolescent, or young adult patients?
◯ Yes
◯ No

If yes, please select which measure(s)
☐ CTCAE
☐ SSPedi
☐ FACT-GOG-NTX
☐ Peds-mTNS
☐ Other

If you use another patient reported outcome measure, please explain
____

In general, how comfortable are you in diagnosing peripheral neuropathy in your pediatric, adolescent, or young adult patients?
◯ Very uncomfortable ◯ Uncomfortable
◯ Neither uncomfortable or comfortable
◯ Comfortable ◯ Very comfortable

In general, how comfortable are you in managing peripheral neuropathy in your pediatric, adolescent, or young adult patients?
◯ Very uncomfortable ◯ Uncomfortable
◯ Neither uncomfortable or comfortable
◯ Comfortable ◯ Very Comfortable

In general, under what circumstances, do you refer your pediatric, adolescent, or young adult patients with neuropathy to physical therapy?
◯ At the first sign of neuropathy
◯ Mild neuropathy ◯ Moderate neuropathy
◯ Severe neuropathy ◯ I do not refer

Please indicate any barriers you encounter when referring patients for physical therapy
____

Please indicate any additional barriers or reasons to not refer (select all that apply)
☐ Physical therapy is not available at my institution ☐ There are insurance barriers
☐ Physical therapy does not have a role in neuropathy diagnosis/management
☐ Physical therapy has a role in neuropathy diagnosis/management, however due to prolonged periods of neutropenia do not recommend patients to receive physical therapy during therapy
☐ Other

If other, please list other barriers	__________________________________
In general, under what circumstances do you start a pharmacologic agent in pediatric, adolescent, or young adult patients with neuropathy?	◯ At the first sign of neuropathy ◯ Mild neuropathy ◯ Moderate neuropathy ◯ Severe neuropathy ◯ I never start a pharmacological agent
Please indicate any barriers you encounter when prescribing a pharmacologic agent	__________________________________
If you do not start a pharmacological agent, please choose from the following	◯ Drugs are not helpful in controlling neuropathic pain ◯ I do not know enough about neuropathy pharmacologic agents to feel comfortable prescribing them ◯ Other
If you do not start a pharmacologic agent, please provide your rationale	__________________________________
Are you a pediatric oncologist or medical oncologist?	◯ Pediatric Oncologist<br>◯ Medical Oncologist<br>◯ Both Pediatric and Medical Oncologist<br>◯ Other
If you are not an oncologist, please state your role in the medical team	__________________________________
How do you describe yourself?	◯ Male<br>◯ Female<br>◯ Trans Male/ Trans Male<br>◯ Trans Female/ Trans Female<br>◯ Genderqueer/ Gender Non- Conforming<br>◯ Different Identify
Years of practice after completion of fellowship?	◯ Less than 5 years<br>◯ 5 to 9 years<br>◯ 10 to 19 years<br>◯ 20 to 30 years<br>◯ More than 30 years
In which type of center do you practice? (Select all that apply)	☐ NCI designated cancer center<br>☐ Academic medical center<br>☐ Private practice affiliated with an academic medical center<br>☐ Private practice not affiliated with an academic medical center<br>☐ Other
Please list other center(s) in which you practice	__________________________________
I primarily care for the following patient populations: (Select all that apply)	☐ Leukemia<br>☐ Lymphoma<br>☐ Neuro-Oncology<br>☐ Solid Tumors
